# Ulcerative Gastritis and Esophagitis in Two Children with *Sarcina ventriculi* Infection

**DOI:** 10.3389/fmed.2017.00145

**Published:** 2017-08-30

**Authors:** Tim G. J. de Meij, Michiel P. van Wijk, Aart Mookhoek, Andries E. Budding

**Affiliations:** ^1^Department of Paediatric Gastroenterology, VU University Medical Centre, Amsterdam, Netherlands; ^2^Department of Pathology, VU University Medical Centre, Amsterdam, Netherlands; ^3^Department of Medical Microbiology and Infection Control, VU University Medical Centre, Amsterdam, Netherlands

**Keywords:** esophagitis, children, microbiota, IS-pro, *Sarcina ventriculi*, gastritis

## Abstract

*Sarcina ventriculi* is a Gram-positive, obligate anaerobic coccus, with a characteristic morphology. Only 22 cases of human infections by this microorganism, including 7 in children, have been reported in literature so far. Affected subjects usually present with abdominal pain, nausea, vomiting, and delayed gastric emptying. However, life-threatening complications, like emphysematous gastritis and gastric perforation have also been described. Gastroparesis and gastric outlet obstruction have been considered as a potential etiologic factor. All pediatric cases described thus far presented with concomitant gastrointestinal pathology, such as *Helicobacter pylori* gastritis, celiac disease, infection with *Giardia lamblia* or *Candida* spp. Here, we report two children with *S. ventriculi* infection, in whom the diagnosis was established by typical histological findings in mucosal biopsies. The first child presented with hematemesis due to ulcerative esophagitis and gastritis, the second child with a history of esophageal stricture had ulcerative gastritis. Confirmation of *S. ventriculi* infection is feasible by molecular microbiota detection methods, since this microorganism cannot be detected by classical culture techniques. Prompt treatment with antibiotics could prevent life-threatening complications.

## Introduction

*Sarcina ventriculi* is a Gram-positive, obligate anaerobic coccus, present in the soil, with a characteristic tetrad or octet morphology. Only 22 cases of human infections by this microorganism, including 7 children, have been described in literature to date. The majority of cases presented with abdominal pain, nausea, vomiting, and delayed gastric emptying. In a few cases, infection was associated with life-threatening complications, like emphysematous gastritis and gastric perforation. Here, we report two children with a *S. ventriculi* infection in whom the diagnosis could be made on typical histological findings in the mucosal biopsies. The first child presented with hematemesis due to ulcerative esophagitis and gastritis, the second child with a history of esophageal stricture had ulcerative gastritis. In both children, symptoms resolved completely following targeted antibiotics.

## Case Presentation

**Figure 1:**
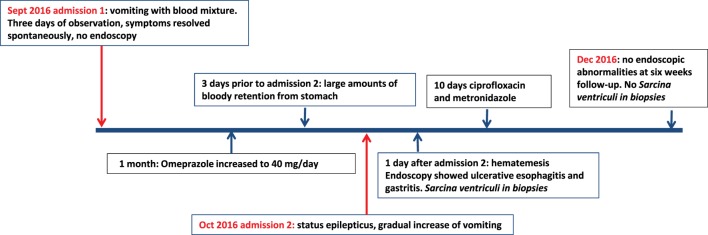
Timeline displaying the course of clinical symptoms of case 1.

The first patient was a 12-year old girl with a history of psychomotor retardation and refractory epilepsy based on West syndrome. Furthermore, she was diagnosed twice with an episode of *Helicobacter pylori*-associated gastritis, successfully eradicated with triple therapy on both occasions. Percutaneous endoscopic gastrostomy (PEG) was performed early in life for long-term administration of enteral nutrition. She was admitted to the hospital because of mild dehydration due to intractable vomiting since 3 days, possibly including blood. Aspiration of stomach content *via* the PEG tube revealed retention of a large amount of brownish fluid. On physical examination, the patient was tachycardic (110 beats/min), with normal blood pressure and oxygen saturation. Palpation of the abdomen was not painful, and no other physical signs were present, besides her pre-existent neurological impairment. Laboratory investigation showed normal hemoglobin concentration (9.5 mmol/l), mean cellular volume 94 fl, normal platelet count, and normal infection and clotting parameters. At admission, she was already on omeprazole (20 mg/day), next to phenobarbital and gabapentin as antiepileptic treatment. Differential diagnosis included Mallory–Weiss syndrome, viral gastro-enteritis, esophagitis, recurrence of *H. pylori*-associated ulcer/gastritis, and bleeding from esophageal varices. Portal hypertension was excluded by abdominal ultrasound and *H. pylori* antigen stool test was negative. Omeprazole was increased to 40 mg/day. No hematemesis was observed during the 3 days of admission, and therefore, esophagogastroduodenoscopy was not performed. After 1 month, the patient was readmitted because of status epilepticus, possibly due to insufficient intake of antiepileptic medication linked to a gradual increase of vomiting. Convulsions were adequately treated with intravenously administered midazolam. Three days prior to this second admission, the mother of the patient had aspirated large amounts of bloody retention from the stomach, but the hemoglobin level was not decreased compared to the previous admission. One day following this second admission, the patient had an episode of hematemesis, and therefore, a esophagogastroduodenoscopy was performed under general anesthesia. A timeline displaying the course of clinical symptoms is depicted in Figure 1.

**Figure 2:**
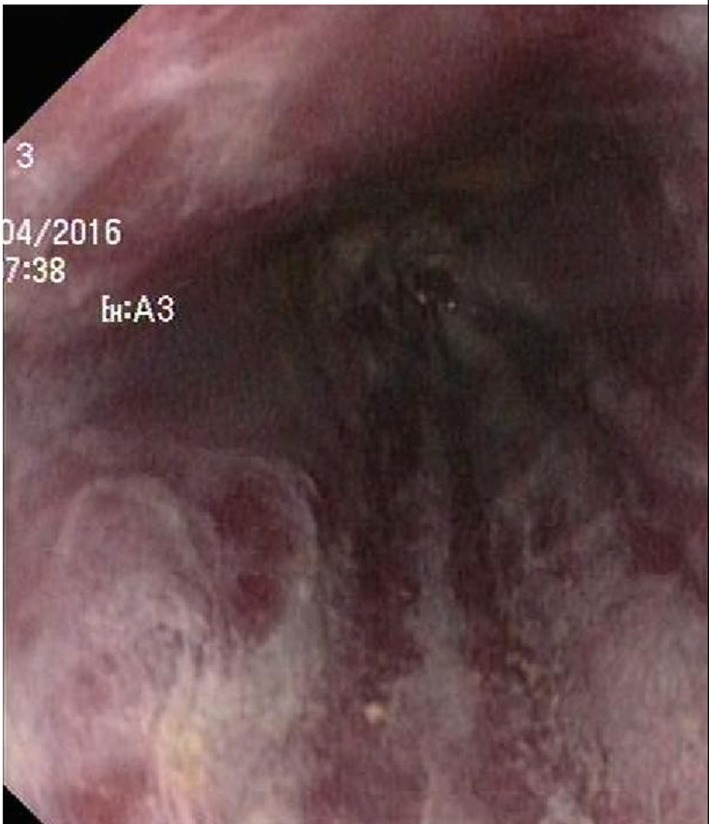
Erosive esophagitis.

**Figure 3:**
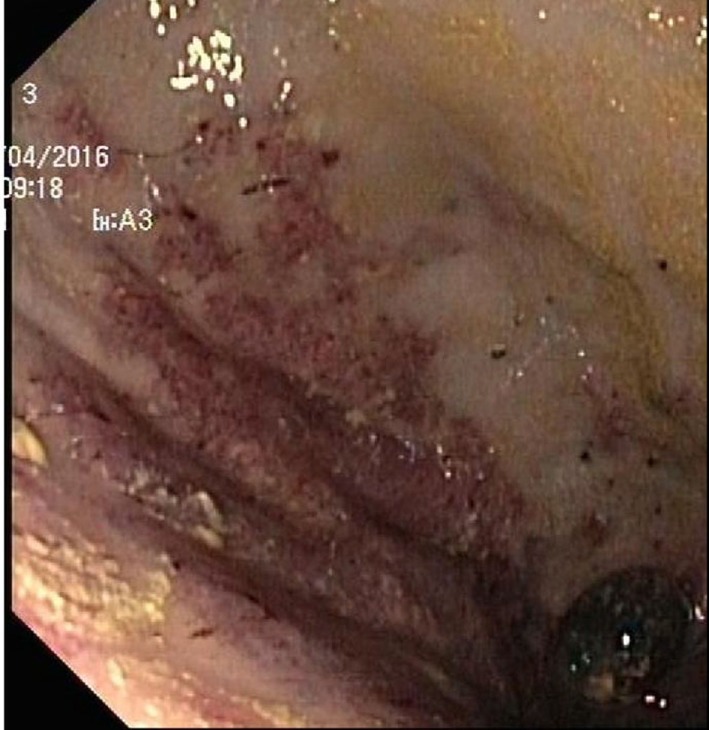
Erosive gastritis.

**Figure 4:**
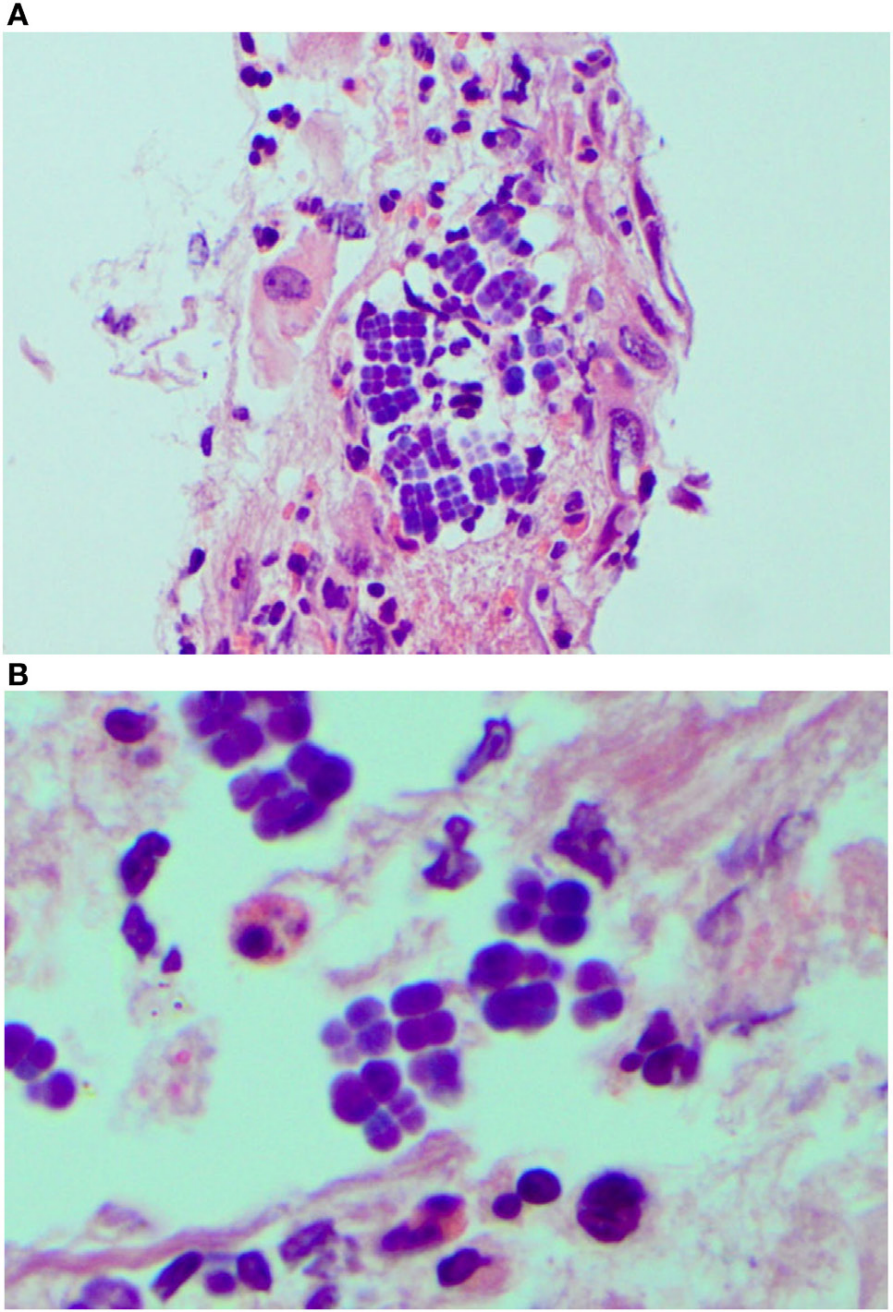
*Sarcina ventriculi* in esophageal biopsies. These microorganisms appear to be arranged in tetrads [**(A)** 200×], but on higher magnification appear to be arranged in cubes composed of eight individual spheres [**(B)** 400×].

Endoscopy showed a severe, erosive esophagitis of the distal 15 cm of the esophagus (Figure 2). Furthermore, a multifocal hemorrhagic antral and corporal gastritis was observed (Figure 3), next to two circular antral ulcers with a diameter of 5 mm. Notably, a large amount of stomach retention was observed, indicative for delayed gastric emptying. Histological examination of esophageal and gastric biopsies showed severe ulcerative esophagitis and gastritis, next to the presence of bacteria in the gastric and esophageal mucous surface. The bacteria were arranged in tetrad and octet groups, compatible with *S. ventriculi* (Figures 4A,B). *H. pylori* was not observed.

Culture of the mucosal biopsies was negative, including culture for *H. pylori*. Subsequently, we applied a molecular microbiota profiling technique, called IS-pro, which confirmed the presence of *S. ventriculi*. Patient was treated with ciprofloxacin and metronidazole for 10 days and the symptoms of bloody stomach retention and hematemesis resolved completely within several days. Control esophagogastroduodenoscopy performed after 6 weeks showed complete healing of the gastric and esopgaheal mucosa. In the mucosal biopies, *S. ventriculi* could no longer be detected. Duration of follow-up was 12 months, during which period no recurrence of symptoms was reported.

### Case 2

**Figure 5:**
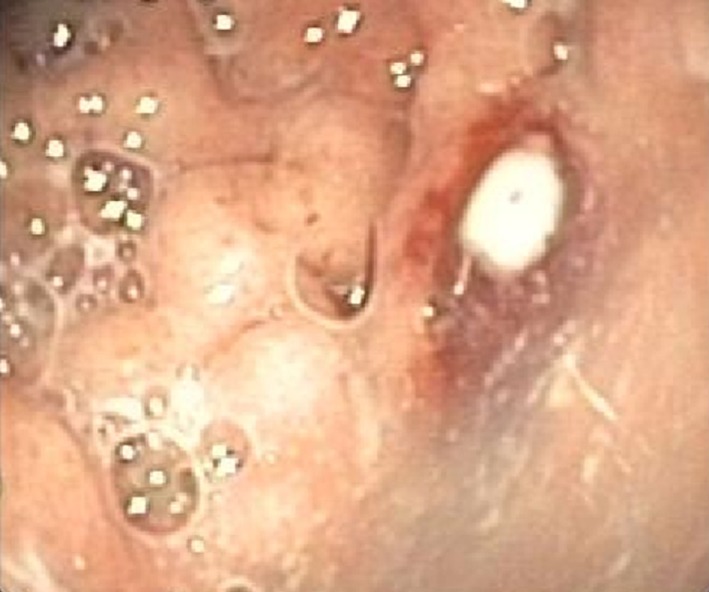
Circular gastric ulcer.

**Figure 6:**
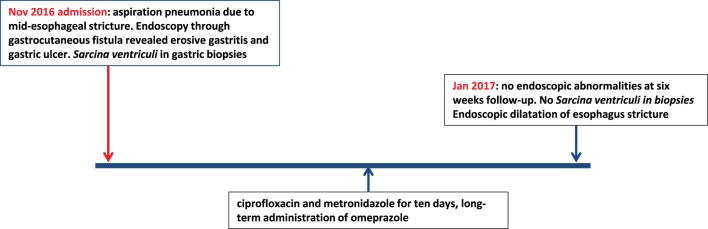
Timeline displaying the course of clinical symptoms of case 2.

The second patient was a 15-year-old Caucasian girl with a history of severe neurological impairment and epilepsy due to intracranial hemorrhage from a cerebral arteriovenous malformation at 8 years of age. She was prescribed Clobazam and Keppra as antiepileptic agents and had a PEG tube for administration of enteral nutrition. She presented with respiratory failure due to an aspiration pneumonia, for which mechanical ventilation was required. Because of the inability to insert a nasogastric tube during admission, a single-contrast study of the esophagus was performed, which showed a mid-esophageal stricture over a length of 10 cm. An upper endoscopy was performed which confirmed the presence of a mid-esophageal pinpoint stenosis. Endoscopy was also performed through the gastrocutaneous fistula for retrograde inspection of the distal esophagus. Here, we observed a diffuse erosive gastritis and a large gastric circular ulcer with a diameter of 10 mm at the gastro-esophageal junction with no signs of esophagitis (Figure 5). Furthermore, a significant amount of stomach retention was seen, despite 12 h of fasting, suggestive of delayed gastric emptying. Histological examination of gastric biopsies showed active gastritis with the presence of microorganisms arranged in tetrads, characteristic of *S. ventriculi*, which was confirmed by the IS-pro technique. *H. pylori* and *Giardia lamblia* were not detected. The patient was prescribed ciprofloxacin and metronidazole for 10 days, next to omeprazole (40 mg/day). At endoscopic follow-up 6 weeks following antibiotics, the gastritis and gastric ulcer were healed completely and *S. ventriculi* could no longer be detected from the biopsies. Endoscopic dilatation of the esophageal stricture was successfully performed using Savary-Gilliard bougies. A timeline with the course of clinical symptoms is depicted in Figure 6. During follow-up period of 8 months, no recurrence was reported.

## Discussion

In 1842, John Goodsir was the first to describe the presence of *Sarcina* microorganisms in the stomach of a patient presenting with abdominal pain, bloating, and vomiting (1). The pathogenic role of *S. ventriculi* has been well established in the veterinary literature, including description of several fatal cases, called “abomasal bloat,” in livestock (2, 3). Its pathogenicity in humans has been questioned until recent years, since only few symptomatic infections with *Sarcina* organisms in humans have been described, only 22 cases in literature so far (4–18), while *S. ventriculi* has also been detected in asymptomatic subjects (19, 20). Sites of infections described thus far include the stomach (85%), esophagus (10%), and duodenum (5%) (21). Presenting symptoms in patients with *S. ventriculi* commonly include abdominal pain, nausea, vomiting, and, as in our case, delayed gastric emptying (8, 9). Severe complications like emphysematous gastritis and gastric perforation have also been reported (4, 5). It has been suggested that impaired gastric emptying could be a risk factor for the growth of *S. ventriculi* (8, 9). The majority of affected patients do not have mucosal injury on endoscopy, but increasing evidence suggests that *S. ventriculi* is associated with gastric ulcers, with an incidence of over 30% in *Sarcina* infections, and subsequently with an increased risk for emphysematous gastritis and gastric perforation (7, 21). Only seven children with symptomatic *S. ventriculi* infection have been described in literature so far (5–7, 11, 12, 22), two of them presenting with (partial) gastric necrosis. Notably, all described children had a concurrent gastrointestinal disease, including *H. pylori* gastritis (*n* = 2), infection with *G. lamblia* or *Candida* spp., history of esophageal atresia and post gastric pull through with anastomotic narrowing, and celiac disease. Our first case had no (recognized) gastrointestinal diagnosis, both cases had a PEG tube placement for administration of enteral nutrition. An association between a PEG tube and *S. ventriculi* infection has not been described previously. In three of seven affected children described in literature, endoscopy revealed the presence of distal esophagitis and none of them had hematemesis as presenting symptom (7, 8). Cultivation of *S. ventriculi* is complicated by its complex nutritional requirements, but diagnosis can usually be made based on typical morphological features (11). Diagnosis can be confirmed by means of molecular techniques like sequencing or, as in our case, by IS-pro, a eubacterial DNA-based molecular detection technique (23). IS-pro is based on identification of species-specific length polymorphisms of the interspacer region and phylum-specific sequence polymorphisms of 16S rDNA. Treatment of *S. ventriculi* usually consists of proton pump inhibitors and antibiotics, with metronidazole and ciprofloxacin as most frequently prescribed agents (3, 19). In our first case description, the patient was already on proton pump inhibitors prior to onset of complaints, and she recovered both clinically and endoscopically following administration of ciprofloxacin and metronidazole.

The association between *S. ventriculi* infections and esophageal strictures, as described in case 2, has already been described over a century ago (22). In an intriguing experiment by Beijerinck et al. performed in 1911 under strict anaerobic conditions to prove the similarity between *S. ventriculi* isolated from garden soil and from stomach contents, the researchers used from stomach contents from patients with esophageal stenosis and suffering from *S. ventriculi* infection (22). This association has not been described in subsequent reports on human *S. ventriculi* infections. In case 2, *S. ventriculi* was detected in mucosal biopsies from the stomach, but not from the esophagus. We, therefore, believe that *S. ventriculi* infection is more likely to be a consequence rather than a cause of esophageal strictures; severe stenosis may create optimal anaerobic conditions for *S. ventriculi* to grow.

In conclusion, *S. ventriculi* infection is a rare cause of ulcerative gastritis and esophagitis in children, with delayed gastric emptying as possible predisposing factor. Diagnosis is usually established by typical histological findings and can be confirmed by means of molecular detection techniques. Since infection may lead to life-threatening complications, it should be treated promptly with proton pump inhibitor and antibiotics.

## Ethics Statement

This study was carried out in accordance with the recommendations of the Ethical Commitee VU University medical center with written informed consent from all included subjects. The parents of these subjects gave written informed consent in accordance with the Declaration of Helsinki. The protocol was approved by the Ethical Commitee VU University medical center.

## Author Contributions

TM is treating physician of described patients and wrote first draft of the manuscript. MW performed the endoscopies and critically assessed the manuscript. AM performed histological analyses and critically assessed the manuscript. AB performed microbiota analysis and critically assessed the manuscript.

## Conflict of Interest Statement

The authors declare that the research was conducted in the absence of any commercial or financial relationships that could be construed as a potential conflict of interest.

## References

[1] Goodsir J History of a case in which a fluid periodically ejected from the stomach contained vegetable organisms of an undescribed from. Edinb Med Surg J (1842) 57:430–43. PMC5791290 30330668

[2] Edwards GT Woodger NG Barlow AM Bell SJ Harwood DG Otter A *Sarcina*-like bacteria associated with bloat in young lambs and calves. Vet Rec (2008) 163:391–3. 10.1136/vr.163.13.391 18820327

[3] Marshall TS. Abomasal ulceration and tympany of calves. Vet Clin North Am Food Anim Pract (2009) 25:209–20. 10.1016/j.cvfa.2008.10.010 19174290

[4] Tuuminen T Suomala P Vuorinen S. *Sarcina ventriculi* in blood: the first documented report since 1872. BMC Infect Dis (2013) 13:169. 10.1186/1471-2334-13-169 23566207 PMC3623782

[5] Tolentino LF Kallichanda N Javier B Yoshimori R French SW A case report of gastric perforation and peritonitis associated with opportunistic infection by *Sarcina ventriculi*. Lab Med (2003) 34:535–7. 10.1309/CDFF04HE9FHDQPAN

[6] Laass MW Pargac N Fischer R Bernhardt H Knoke M Henker J Emphysematous gastritis caused by *Sarcina ventriculi*. Gastrointest Endosc (2010) 72:1101–3. 10.1016/j.gie.2010.02.021 20538273

[7] Lam-Himlin D Tsiatis AC Montgomery E Pai RK Brown A Razavi M *Sarcina* organisms in the gastrointestinal tract: a clinicopathologic and molecular study. Am J Surg Pathol (2011) 35:1700–5. 10.1097/PAS.0b013e31822911e6 21997690 PMC3193598

[8] Sauter JL Nayar SK Anders PD D’Amico M Butnor KJ Wilcox RL Co-existence of *Sarcina* organisms and *Helicobacter pylori* gastritis/duodenitis in pediatric siblings. J Clin Anat Pathol (JCAP) (2013) 1:103. 25664331 PMC4318520

[9] Ratuapli SK Lam-Himlin DM Heigh RI. *Sarcina ventriculi* of the stomach: a case report. World J Gastroenterol (2013) 19:2282–5. 10.3748/wjg.v19.i14.2282 23599657 PMC3627895

[10] Louis GB Singh P Vaiphei K *Sarcina* infection. BMJ Case Rep (2014) 2014:bcr2013201185 10.1136/bcr-2013-201185 PMC3902398 24419638

[11] Kumar M Bhagat P Bal A Lal S. Co-infection of *Sarcina* and *Giardia* in a child. Oxf Med Case Reports (2014) 2014:118–9. 10.1093/omcr/omu046 25988051 PMC4370029

[12] Karakus E Kirsaclioglu CT Coincidence of celiac disease with *Sarcina* infection. Turk J Gastroenterol (2014) 25(Suppl 1):318 10.5152/tjg.2014.8028 25910360

[13] Bhagat P Gupta N Kumar M Radotra BD Sinha SK. A rare association of *Sarcina* with gastric adenocarcinoma diagnosed on fine-needle aspiration. J Cytol (2015) 32:50–2. 10.4103/0970-9371.155238 25948948 PMC4408681

[14] Carrigan S Grin A Al-Haddad S Iakovlev V Streutker C Moore T Emphysematous oesophagitis associated with *Sarcina* organisms in a patient receiving anti-inflammatory therapy. Histopathology (2015) 67:270–2. 10.1111/his.12599 25410912

[15] Berry AC Mann S Nakshabendi R Kanar O Cruz D Gastric *Sarcina ventriculi*: incidental or pathologic? Ann Gastroenterol (2015) 28:495. 26424003 PMC4585398

[16] Chougule A Muthu V Bal A Rudramurthy SM Dhooria S Das A Pulmonary gangrene due to *Rhizopus* spp., *Staphylococcus aureus, Klebsiella pneumoniae* and probable *Sarcina* organisms. Mycopathologia (2015) 180:131–6. 10.1007/s11046-015-9904-3 26022794

[17] Sopha SC Manejwala A Boutros CN. *Sarcina*, a new threat in the bariatric era. Hum Pathol (2015) 46:1405–7. 10.1016/j.humpath.2015.05.021 26198746

[18] Medlicott SAC Adams F *Sarcina ventricularis* complicating a patient status post vertical banded gastroplasty: a case report. J Gastroenterol Hepatol Res (2015) 14:1481–4. 10.17554/j.issn.2224-3992.2015.04.498

[19] Haroon Al Rasheed MR Kim GJ Senseng C A rare case of *Sarcina ventriculi* of the stomach in an asymptomatic patient. Int J Surg Pathol (2015) 24(2):142–5. 10.1177/1066896915610196 26453674

[20] Ferrier D The constant occurrence of *Sarcina ventriculi* (Goodsir) in the blood of man and the lower animals: with remarks on the nature of sarcinous vomiting. Br Med J (1872) 1(578):98–9. 10.1136/bmj.1.578.98 PMC2297174 20746505

[21] Gaspar BL. The significance of *Sarcina* in routine surgical pathology practice. APMIS (2016) 124(6):436–43. 10.1111/apm.12526 26918758

[22] Canale-Parola E Wolfe R Studies on *Sarcina ventriculi*. I. Stock culture method. J Bacteriol (1960) 79:857–9. 13807366 10.1128/jb.79.6.857-859.1960 PMC278791

[23] Budding AE Grasman ME Lin F Bogaards JA Soeltan-Kaersenhout DJ Vandenbroucke-Grauls CM IS-pro: high-throughput molecular fingerprinting of the intestinal microbiota. FASEB J (2010) 24(11):4556–64. 10.1096/fj.10-156190 20643909

